# Helium beam shadowing for high spatial resolution patterning of antibodies on microstructured diagnostic surfaces

**DOI:** 10.1186/1559-4106-8-9

**Published:** 2013-04-03

**Authors:** Eliedonna Cacao, Tim Sherlock, Azeem Nasrullah, Steven Kemper, Jennifer Knoop, Katerina Kourentzi, Paul Ruchhoeft, Gila E Stein, Robert L Atmar, Richard C Willson

**Affiliations:** 1 Department of Chemical and Biomolecular Engineering, University of Houston, Houston, TX, USA; 2 Department of Electrical and Computer Engineering, University of Houston, Houston, TX, USA; 3 Department of Biomedical Engineering, University of Houston, Houston, TX, USA; 4 Materials Engineering, University of Houston, Houston, TX, USA; 5 Baylor College of Medicine, Houston, TX, USA; 6 Department of Biology and Biochemistry, University of Houston, Houston, TX, USA; 7 The Methodist Hospital Research Institute, Houston, TX, USA

## Abstract

**Abstract:**

We have developed a technique for the high-resolution, self-aligning, and high-throughput patterning of antibody binding functionality on surfaces by selectively changing the reactivity of protein-coated surfaces in specific regions of a workpiece with a beam of energetic helium particles. The exposed areas are passivated with bovine serum albumin (BSA) and no longer bind the antigen. We demonstrate that patterns can be formed (1) by using a stencil mask with etched openings that forms a patterned exposure, or (2) by using angled exposure to cast shadows of existing raised microstructures on the surface to form self-aligned patterns. We demonstrate the efficacy of this process through the patterning of anti-lysozyme, anti-Norwalk virus, and anti-*Escherichia coli* antibodies and the subsequent detection of each of their targets by the enzyme-mediated formation of colored or silver deposits, and also by binding of gold nanoparticles. The process allows for the patterning of three-dimensional structures by inclining the sample relative to the beam so that the shadowed regions remain unaltered. We demonstrate that the resolution of the patterning process is of the order of hundreds of nanometers, and that the approach is well-suited for high throughput patterning.

## Background

Creating patterned biological functionality of antibodies, enzymes, or cell-adhesion molecules is an essential tool for the development of high-performance bioanalytical devices and diagnostics. Patterned antibody surfaces have previously been formed by ultraviolet (UV) [1–3] and electron beam [4–6] exposure of polymeric films, followed by a development step to create two chemically-distinct surfaces which can be selectively functionalized. These approaches take advantage of well-established lithographic techniques and can achieve very high spatial resolution on planar substrates. Stamping techniques also have been developed to transfer chemically-orthogonal self-assembled monolayers (SAMs) to surfaces by inking a stamp, typically made of polydimethylsiloxane, with the SAM molecule and transferring it from the protrusions on the stamp directly onto the substrate [7, 8]. Direct “writing” of SAMs using an AFM tip has also been demonstrated [9, 10], and nanopipette delivery of biomolecules to specific areas of a previously etched surface also has been developed [11–13]. While these techniques are well established and extremely useful, none are well-suited for patterning surfaces with three-dimensional structures without the need for precise alignment with the existing patterns; an approach to this problem is the subject of the present work.

**Figure 1:**
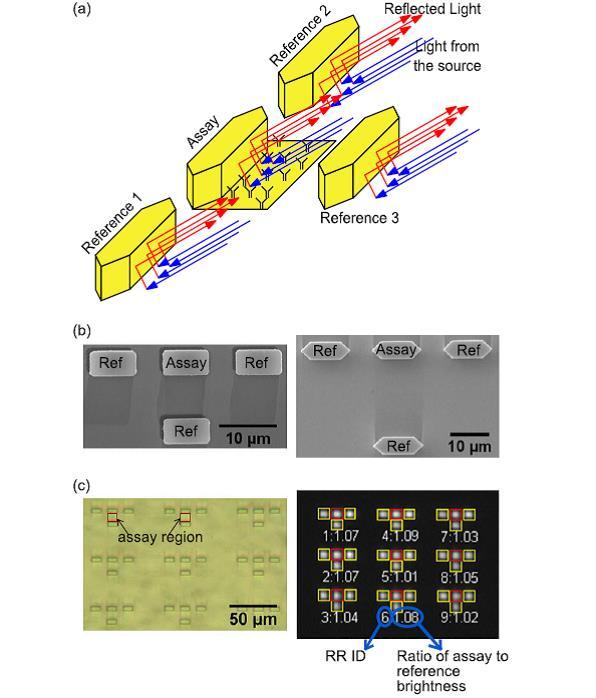
**Micron-scale retroreflector-based read-out.** (**a**) A schematic of a retroreflector-based readout with micron-scale sensing areas, where the brightness of light reflected from the central reflector is modulated by the analyte-driven assembly of scattering elements. The readout relies on detecting an intensity change relative to the three always-bright reference reflectors, and, thus, the ability selectively to localize the antibodies in front of the central reflector is important and the motivation for this work. (**b**) Scanning electron microscope images of the first-generation (left) and the second-generation (right) reflectors used in this work. (**c**) A top-down optical microscope image (left) and the corresponding retroreflector (RR) optical readout (right) of nine sets of four reflectors, where the a group identification number (i.e., four reflectors, or a *tetrad*) and the gray-scale ratio of assay signal to the average of the three reference signals for each group of four reflectors are shown. For the optical readout, the camera is tilted about 35 degrees.

We are developing a biosensing platform in which the brightness of microfabricated retroreflecting structures is modulated in the presence of analyte by capture of opacifying elements, especially magnetic sample-prep particles. To simplify readout, we form reference retroreflectors proximal to assay reflectors so that the brightness of these structures can be compared in a single image frame to monitor changes in the assay region. The schematic in Figure 1a shows three-dimensional retroreflective protrusions that reflect light back to its source.

The structures consist of two perpendicular, mirrored surfaces so that light entering the structures reflects from both surfaces to return to its source. The more common retroreflecting design that is used in street and sign markings consist of three mirrored surfaces, which allows them to appear bright for a wide range of azimuthal orientations; the structures used in this work retroreflect only for a fixed azimuth but over a wide range of altitudes, requiring alignment in one direction. The image that is formed consists of four bright spots, each corresponding to the reflections from the longer walls of the structures.

With this design, the three outer reflectors produce an always-bright reference signal for simple identification by automated image recognition algorithms and normalization of the reflectivity of the central assay reflector, which is responsive to analyte. Figure 1b shows scanning electron microscope (SEM) images of first-generation rectangular retroreflectors (left), and second-generation tapered structures (right). The second-generation geometry was designed to experience lower shear forces when fluid flows in the horizontal direction across the structure while still reflecting light from the longer sidewalls. In the presence of the target, the assay reflector brightness decreases when the analyte captured in the assay region (left-hand image in Figure 1c) is labeled with effective light scattering structures that attenuate the reflected signal. Automated image analysis techniques can identify the structures and calculate the ratio of the assay reflector brightness to those of the references, as illustrated in the right-hand image of Figure 1c. The ratio of the intensity of the assay (central reflector) region to the average of the three reference regions is shown alongside a region identifier.

We therefore are interested in developing a patterning process that can (1) take advantage of the presence of three-dimensional structures to avoid the need to align a second pattern to the existing structures and (2) pattern surfaces with a large degree of topography and with high throughput. To address this problem, we have developed a method for casting shadows of a broad, energetic (5–7 keV) helium ion beam and for using this beam to locally modify the activity of a surface uniformly coated with antibodies. Because of the wavelength of these particles is extremely small, this approach has a very large depth of field and can cast high quality shadows over long distances.

## Methods

### Antibodies

Polyclonal rabbit anti-*Escherichia coli* (*E. coli*) antibodies (20-ER13) were from Fitzgerald Industries International (Acton, MA) and polyclonal rabbit anti-Norwalk virus-like particle (NVLP) antibodies were provided by one of the authors (RLA) [14]. Polyclonal goat anti-rabbit (R2004) and polyclonal goat anti-mouse (M8645) antibodies were purchased from Sigma-Aldrich (St. Louis, MO). Goat anti-mouse antibodies conjugated with alkaline phosphatase (DC05L) were from Calbiochem (EMD Biosciences, Gibbstown, NJ), and monoclonal mouse anti-lysozyme antibodies [15] (HyHEL-5) were prepared for us by Biovest (Minneapolis, MN). All biotinylated antibodies were prepared using the EZ-Link Sulfo-NHS-LC-Biotinylation Kit (21435, Pierce Protein Research Products, Thermo Scientific, Rockford, IL).

### Surface preparation

The retroreflector structures were formed using methods similar to those described in ref. [16]. First, 100 mm diameter silicon wafers were coated with a 5 *μ*m layer of polyimide (Durimide 10/32, Fujifilm Electronic Materials, North Kingstown, RI) by spin-casting (1000 rpm for 2 minutes), and the sample was baked at 350°C for 60 minutes on a hot plate. Next, a 300 nm thick layer of poly(methyl glutarimide) (PMGI) (SF-6 from Microchem Inc., Newton, MA) was also deposited by spin-casting (2000 rpm for 2 minutes) and baked at 180°C for 5 minutes, followed by a 200 nm thick layer of poly(methyl methacrylate) (PMMA) (Microchem Inc., Newton, MA) (2000 rpm for 1 minute), and baked at 180°C for 60 minutes.

The PMMA layer was patterned by electron beam lithography to form the retroreflector structures and developed for 30 seconds in a 1:3 solution of methyl isobutyl ketone and isopropanol (MIBK:IPA), which dissolves the exposed PMMA, and rinsed in IPA before drying in nitrogen. The PMGI was etched in 2.3% tetramethyl ammonium hydroxide (TMAH) in water for 20 seconds, and a 140 nm thick layer of nickel was deposited on the wafer by thermal evaporation. The PMMA was then dissolved in acetone, which removed the nickel in all regions of the wafer, except where the PMMA was patterned. An oxygen reactive ion etch step in a magnetically-enhanced, capacitively-coupled plasma reactor (1 mTorr and 30 watts) transferred the patterns into the polyimide layer with near-vertical sidewalls. Lastly, the structures were coated with a 100 nm thick layer of conformal gold by thermal evaporation.

These gold surfaces were alkanethiol SAM functionalized using the homobifunctional, thiol-cleavable cross-linker DSP (dithiobis-succinimidyl propionate) which has been employed in many biomedical applications [17–19]. The sulfur in the thiol group has strong specific interaction with gold while the N-hydroxy-succinimide (NHS) ester reacts with primary amines present in antibodies. Surfaces were first cleaned by dipping them in anhydrous ethanol for at least 2 minutes, then thoroughly rinsed with deionized water (18 M *Ω*), and finally dried with a stream of compressed nitrogen. The clean surfaces were then immersed into a 10 mM solution of DSP (22585, Pierce Protein Research Products, Thermo Scientific, Rockford, IL) in dimethylsulfoxide, DMSO (472301, >99.9%, ACS reagent, Sigma-Aldrich, St. Louis, MO) and were allowed to incubate for at least 30 minutes at room temperature. Afterwards, the surfaces were washed once with DMSO, then twice with DI water. Protein solution (at least 1 mg/mL in phosphate-buffered saline, 1 × PBS) was immediately spotted on the surfaces and allowed to incubate for at least 2 hours at room temperature. Finally, surfaces were washed twice with 1 × PBS, then thrice with DI water.

### Helium-beam exposure of antibody-functionalized surfaces

A mixed beam of helium ions and atoms was generated by a saddle-field source based on the designs in refs. [20, 21]. The source is mounted onto a vacuum chamber and helium gas is introduced at a pressure of a few mTorr. When a high-voltage DC signal (3–10 kV) is applied, electrons are trapped along oscillating paths through the center of the source and can collide with the gas molecules to ignite a plasma. A fraction of the ions can leave the source with an energy of about 70% of source power supply potential, and some of these ions are neutralized when colliding with background gasses by charge-exchange collisions [22]. The beam, now consisting of ions and atoms, then drifts to an exposure chamber located about 1.5 m from the source. A mechanical shutter allows for automated timing of exposures, and the sample is placed on a motorized *x*−*y* stage that allows for step-and-repeat patterning of larger surfaces or for the patterning of a number of individual samples with varying doses. A mask can be placed in front of the sample to form a pre-defined nano-scale pattern or the sample can be placed on a wedge-shaped sample holder to expose it to the beam at a specific angle.

**Figure 2:**
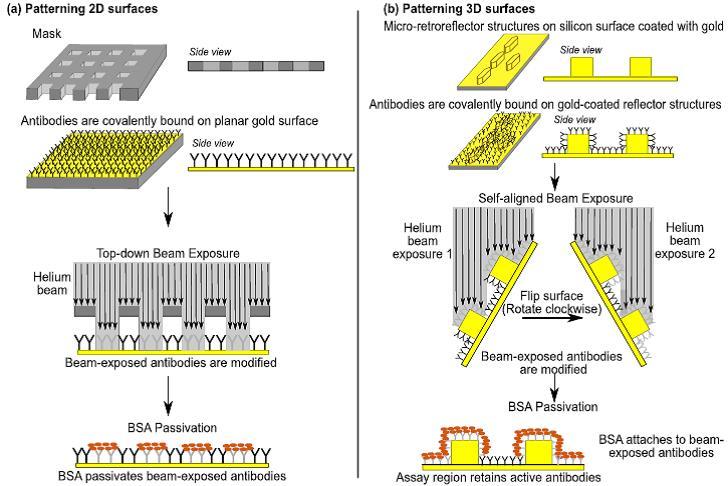
**Protein patterning by helium-beam antibody modification followed by BSA passivation.** (**a**) A top-down beam exposure through a mask allows for selective inactivation of the transparent regions of the mask, while (**b**) a self-aligned beam exposure of the micro-retroreflector structures allowed for formation of patterns in the regions where shadows are cast.

Patterning of proteins on surfaces was accomplished by casting shadows either using a mask in proximity to the surface (Figure 2a) or using the 5 *μ*m tall retroreflector structures on surfaces placed at an angle to the beam (Figure 2b). The ion source discharge was operated at 6 kV and 0.5 mA, which generated a beam with an equivalent current density of about 70 nA/cm^2^, and the exposure time was varied from 15 seconds to 15 minutes, corresponding to a dose range of about 1 to 75 *μ*C/cm^2^. After beam exposure, most surfaces were further passivated with 4% bovine serum albumin, BSA (A7906, Sigma-Aldrich, St. Louis, MO) in 1 × PBS for at least 1 hour at room temperature with mixing on an orbital shaker, except for those surfaces used for studying beam-induced antibody modification.

### Detection and visualization of proteins

Patterned antibodies were visualized using three methods that have been utilized in various biomolecular assays,

1. enzyme-mediated formation of diformazan precipitates by alkaline phosphatase (AP) from its substrate bromochloroindoyl phosphate-nitroblue tetrazolium BCIP-NBT,[23–25],
2. formation of silver nanoparticles by horseradish peroxidase (HRP) EnzMet *silver staining kit*[26–29], and
3. binding of gold nanoparticle probes (colloidal gold conjugated with antibodies) [30–32] or gold nanoparticle-nucleated *silver enhancement*[33–35].

Localized formation of diformazan precipitates used AP conjugates (avidin-conjugated AP, 21321, Pierce Protein Research Products, Rockford, IL) and BCIP-NBT (72091, Sigma-Aldrich, St. Louis, MO). Gold nanoparticle probes (20-PC-40 or 20-PC-100, both from Nanopartz Inc., Loveland, CO) were conjugated with antibodies according to the manufacturer’s protocol. Gold nanoparticle-nucleated silver enhancement used streptavidin-gold conjugates (Nanogold^TM^, 2016, Nanoprobes, Yaphank, NY). The *silver staining kit* consisted of HRP-mediated silver enhancement utilizing streptavidin-polyHRP conjugates (65R-S105PHRP, Fitzgerald Industries International, Acton, MA) and HRP silver staining solution (EnzMet^TM^, 6010, Nanoprobes Inc., Yaphank, NY). The *silver enhancement* solution was composed of 6 mM silver acetate (85140, Sigma-Aldrich, St. Louis, MO) in DI water and 22.5 mM hydroquinone (H9003, Sigma-Aldrich, St. Louis, MO) in citrate buffer (0.14 mM citric acid, 0.09 mM sodium citrate, pH 3.8). Surfaces were incubated with detection conjugates for 1 hour by mixing on an orbital shaker at room temperature, and patterns were formed through a staining step lasting 30 minutes to 1 hour for formation of diformazan precipitates, at least 18 hours for the binding of gold nanoparticle probes, 10 to 15 minutes for gold nanoparticle-nucleated silver enhancement and 2 minutes for each reagent in HRP-mediated silver staining.

For sandwich assays, *E. coli* bacteria (K12 JM109), Norwalk virus-like particles (NVLP), and hen egg white lysozyme (L6876, Sigma-Aldrich, St. Louis, MO) were used as antigen targets. Targets were captured on the surface by immersing the antibody-coated sample in a solution containing the antigen, and incubating at least 1 hour (12 hours for bacteria) at room temperature while mixing on an orbital shaker. After capture, surfaces were washed twice with 1 × PBS followed by the detection methods described in the previous paragraph.

## Results and discussion

### Patterned antibody inactivation through stencil masks

**Figure 3:**
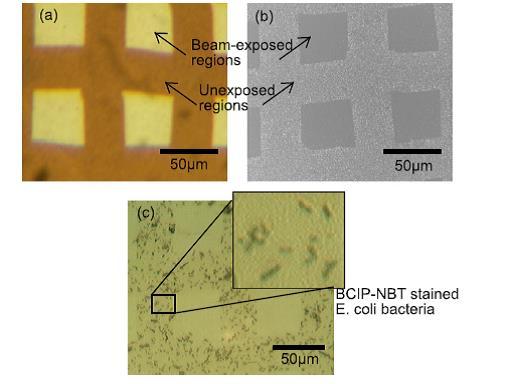
**Images of antibody-coated surfaces patterned by helium beam exposure through a 45*μ*m-mesh mask.** (**a**) An optical microscope image of patterned monoclonal HyHEL-5 antibodies directly detected with goat anti-mouse antibodies conjugated to alkaline phosphatase and BCIP-NBT substrate. (**b**) SEM image of patterned biotinylated polyclonal goat anti-rabbit antibodies directly detected using silver staining of streptavidin-gold conjugates. (**c**) An optical microscope image of BCIP-NBT stained *E. coli* bacteria captured on patterned polyclonal anti-*E.coli* antibodies on a gold surface.

We tested the patterned deactivation procedure on planar surfaces by exposing samples through a stencil mask consisting of 45 *μ*m openings on a 90 *μ*m pitch, as shown schematically in Figure 2a. Gold planar surfaces were covalently coupled with both monoclonal (mouse HyHEL-5) and polyclonal (biotinylated goat anti-rabbit) antibodies, exposed to the helium beam, and dipped in the BSA solution to passivate the exposed areas. In Figure 3a, patterned HyHEL-5 murine monoclonal antibodies were detected by specific binding of secondary antibodies (anti-mouse antibodies conjugated with alkaline phosphatase) and visualized by conversion of BCIP-NBT to diformazan precipitates. This optical image shows that the precipitate accumulates on the beam-protected/unexposed regions (the dark areas in the image), while the exposed regions showed no precipitate (light areas). A similar result was obtained for polyclonal antibodies, as shown in an SEM image in Figure 3b, where the biotinylated goat anti-rabbit antibodies were detected by silver staining of streptavidin-gold conjugates. The image shows that the unexposed regions form silver deposits, as the antibodies remained functional, while the exposed regions were fully passivated by the BSA.

Antibody capture of bacteria showed similar results. In this experiment, a surface bearing immobilized polyclonal anti-*E. coli* antibodies was patterned using the same mask and was dipped into the BSA solution. After enzyme capture and BCIP-NBT staining, the bacteria were seen in the unexposed regions, as is shown in Figure 3c. This result again confirms that antibodies protected from the helium beam exposure retained their functionality and shows that micron-scale bacteria can be selectively captured on the unexposed regions. Moreover, the darkening is observed only on the surface of the bacterium, not on the surfaces adjacent to the bacteria, making the bacterium itself a distinctive light-scattering object that can be detected by the facile automated observation of a single retroreflector structure.

### Patterning of 3D structures

**Figure 4:**
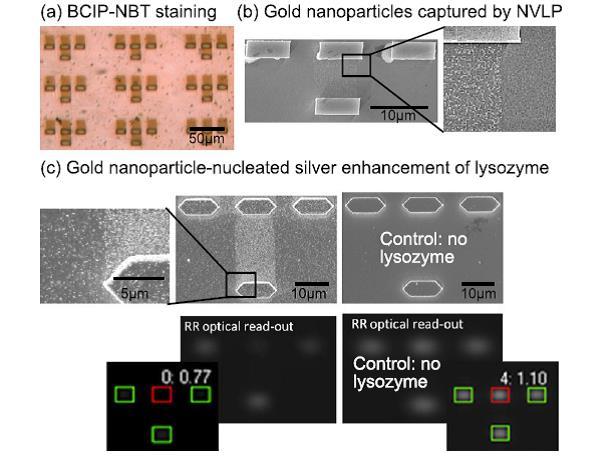
**Antibody patterning by self-aligned helium beam exposure of micro-retroreflector tetrad.** (**a**) Optical microscope image of patterned polyclonal goat anti-mouse antibodies directly detected by biotinylated mouse HyHEL-5 antibodies, avidin-alkaline phosphatase conjugates and BCIP-NBT substrate. (**b**) SEM image of 40 nm gold particles conjugated with anti-NVLP antibodies held down by NVLP captured on patterned polyclonal anti-NVLP antibodies. (**c**) SEM images of biotinylated lysozyme captured on patterned HyHEL-5 antibodies detected using nucleation by silver staining of streptavidin-gold conjugates, and their corresponding retroreflector (RR) optical read-out, which is the ratio of the brightness of the assay reflector and the average of the three reference reflectors.

To form the patterned retroreflector readout illustrated in Figure 1a, samples were exposed to the helium beam at an angle, as shown in the schematic diagram in Figure 2b. For completeness, this would be done from two angles to ensure that both the front and the back of the structures are properly passivated. During the development work described here, however, surfaces were exposed at only one angle to speed processing, and so the areas behind the retroreflectors still have active antibodies. This can be seen in the tails behind the two top reference reflectors and the assay reflector in every tetrad shown in Figure 4a, where polyclonal anti-mouse antibodies protected from the beam were visualized through the localized formation of diformazan precipitates.

Figure 4b and Figure 4c show the detection of Norwalk virus-like particles (NVLP) and lysozyme, respectively. Selective binding of gold nanoparticle labels (conjugated with polyclonal anti-NVLP antibodies) in the assay region was observed, which confirms the presence of captured Norwalk VLPs. Successful capture of biotinylated lysozyme (pre-mixed with streptavidin-gold conjugates) was confirmed by gold nanoparticle-nucleated silver enhancement. As observed in the retroreflector (RR) optical read-out, which is also shown with the overlaid relative intensities, localized deposition of silver nanoparticles darkened the assay reflector by about 30% (i.e., the ratio of assay reflector brightness to the average reference reflectors changed from 1.10 to 0.77 for the control and the silver stained samples, respectively). Figure 4c also shows that nonspecific silver particles were formed on areas other than the assay region, which is attributed to the nonspecific binding of either lysozyme or streptavidin-gold conjugates and not to silver formation in solution, as the control surface (silver-stained, but without lysozyme-nanoparticle complexes) had very few silver particles. This nonspecific silver formation also slightly darkens the reference reflectors, but does not significantly affect the read-out. This is because the reported values are ratios of the assay reflector brightness to the average brightness of three reference reflectors. This inherent self-calibration minimizes the effect of any nonspecific staining and improves the reliability of the assay.

### Chemical modification mechanism

While the process of forming reference and assay detectors works well, it was not clear what the mechanism for the passivation of the exposed surfaces is. In order to help understand this, the following sets of experiments, which employ pattering through a stencil mask, were designed: HyHEL-5 monoclonal antibodies were coupled to a gold-coated silicon surface via the DSP crosslinker and then exposed to helium beam through a stencil mask with 70 *μ*m openings on a 110 *μ*m pitch. After beam exposure, the surfaces were

**Figure 5:**
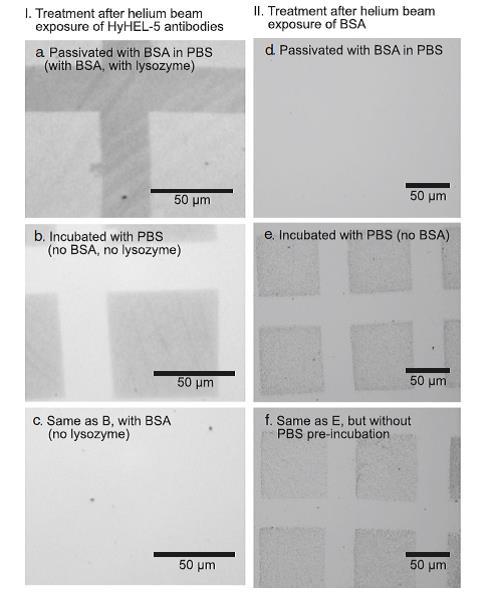
**Effects of helium beam modification of antibody and BSA, and BSA passivation: protein patterning by helium-beam exposure through a mask with 70*μ*m openings and 110*μ*m spacing.** (**I**) Optical microscope images of patterns formed by gold nanoparticle-nucleated silver enhancement on surfaces (**a**) passivated with BSA after beam exposure and incubated with biotinylated lysozyme followed by streptavidin-gold conjugate, or (**b**) incubated with PBS (no BSA and no lysozyme) after beam exposure and incubated with streptavidin-gold conjugate, or (**c**) passivated with BSA (no lysozyme) after beam exposure and incubated with streptavidin-gold conjugate. (**II**) Optical microscope images of patterns formed by 100 nm gold nanoparticle probes on surfaces (**d**) passivated with BSA after beam exposure, or (**e**) incubated with PBS (no BSA) after beam exposure, or (**f**) no PBS pre-incubation.

1. dipped into BSA solution, then incubated with biotinylated lysozyme followed by streptavidin-gold conjugate (Figure 5a),
2. immersed in PBS buffer (with no BSA and no lysozyme) and incubated with streptavidin-gold conjugate (Figure 5b), or
3. dipped into BSA solution (no lysozyme) and incubated with streptavidin-gold conjugate (Figure 5c).

All surfaces were then silver-stained. Figure 5a shows a pattern similar to those presented in Figure 3, where the darkening of the unexposed, protected areas signifies the localized formation of silver nanoparticles while the antibodies in the exposed regions were destroyed, and the chemically-activated surfaces effectively passivated by BSA (light areas). A negative tone pattern was observed in Figure 5b where only the exposed regions showed localized unselective capture of silver nanoparticles (dark areas) while the protected areas remained unreactive. This indicates that, in the absence of BSA, the exposed regions show favorable binding to the streptavidin-gold conjugate. Figure 5c again verifies that exposed regions were well-passivated by BSA since it prevented the subsequent attachment of streptavidin-gold conjugate on these areas.

In the second column of Figure 5, surfaces that were “decorated” with BSA via the DSP crosslinker were exposed to the helium beam using the same mask, and patterns were detected and visualized using 100 nm gold nanoparticles conjugated with polyclonal anti-mouse antibodies. No pattern was observed in Figure 5d, which indicates that the unexposed regions (beam-protected BSA) and beam-exposed areas (further incubated with BSA) were both well-passivated. As shown in Figure 5e, in the absence of BSA after beam exposure, gold nanoparticle probes accumulated on the exposed regions, giving the same pattern as in Figure 5b. This again suggests that beam-exposed regions are reactive towards gold nanoparticle-protein conjugates. Moreover, even without the PBS pre-incubation, gold nanoparticle probes still assembled on beam-exposed regions as shown in Figure 5f. These results indicate that the high contrast shown in Figure 3 and Figure 4 is due to BSA passivating the beam-exposed regions, which are highly active in nonselective capture of proteins when unpassivated.

### Spatial resolution

**Figure 6:**
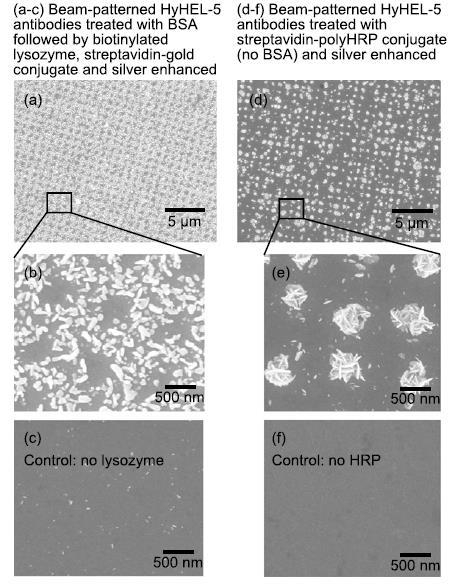
**Evaluation of the resolution of the system by helium-beam exposure of antibody-coated surfaces through a mask containing 300nm diameter openings.** (**a-b**) SEM images of patterns formed by beam exposure of HyHEL-5 antibodies followed by BSA passivation, then treated with biotinylated lysozyme followed by streptavidin-gold conjugate and silver enhancement. (**d-e**) SEM images of patterns formed by beam exposure of HyHEL-5 antibodies followed by PBS (no BSA), then treated with streptavidin-polyHRP conjugate and silver enhancement. (**c**) and (**f**) are images are the corresponding control surfaces.

To evaluate the spatial resolution of this process, a stencil mask containing 300 nm openings with 1 *μ*m spacing was used. The results are shown in Figure 6, where HyHEL-5 antibodies were covalently immobilized on planar gold surfaces via the DSP crosslinker and exposed to the beam through this mask. After exposure, one sample was immersed in BSA solution, followed by addition of biotinylated lysozyme and then streptavidin-gold conjugate (Figure 6a) while another sample was dipped in streptavidin-polyHRP conjugate solution (Figure 6b). Both of these samples were then silver-stained. Figure 6a shows that patterns were formed by the silver nanoparticles in areas where antibodies were protected from the beam. These patterned antibodies were still active as is evidenced by the captured biotinylated lysozyme detected through streptavidin-gold conjugate and visualized by gold nanoparticle-nucleated *silver enhancement*. Figure 6b shows similar results as before in that the exposed regions non-specifically capture protein, and demonstrates the very high spatial resolution of this approach to surface patterning.

## Conclusions

In this study, we have demonstrated the use of a high-throughput collimated helium beam to pattern antibody-coated surfaces (1) through a stencil mask and (2) by using the geometry of existing three dimensional retroreflector structures to form self-aligned patterns. The results show that, when the exposed surfaces are passivated with BSA after beam exposure, there is an extremely high contrast between the exposed and the protected regions of antibody-modified surfaces. While the exposed regions are well-passivated, the beam-protected (unexposed) antibodies remain functional and allowed for the detection of lysozyme, Norwalk virus-like particles and *E. coli* bacteria. We have also established that helium beam exposure modifies the antibodies (and other proteins, in this case, BSA) such that they readily capture other proteins (such as BSA for passivation, but also streptavidin and secondary antibodies for contrasting functionality). Helium beam exposure followed by BSA passivation of antibody-modified surfaces can be used to pattern active antibodies with a resolution of at least 300 nm. The localized passivation of antibodies is also well-suited for forming retroreflecting sensor elements whose relative brightness indicates the presence of a scattering material and which can be used as sensitive readouts for bioassays.
